# PRMT5 regulates the polysaccharide content by controlling the splicing of thaumatin-like protein in *Ganoderma lucidum*

**DOI:** 10.1128/spectrum.02906-23

**Published:** 2023-10-26

**Authors:** Rui Liu, Zhengyan Yang, Tao Yang, Zi Wang, Xin Chen, Jing Zhu, Ang Ren, Liang Shi, Hanshou Yu, Mingwen Zhao

**Affiliations:** 1 Key Laboratory of Agricultural Environmental Microbiology, Ministry of Agriculture; Microbiology Department, College of Life Sciences, Nanjing Agricultural University, Nanjing, China; University of Natural Resources and Life Sciences Vienna, Wien, Austria

**Keywords:** PRMT5, alternative splicing, thaumatin-like protein, *G. lucidum *polysaccharide

## Abstract

In the current study, the silencing of protein arginine methyltransferase 5 (PRMT5) decreased the polysaccharide content of *Ganoderma lucidum* compared to that of the wild-type (WT) strain. Furthermore, RNA-seq screening showed that the glycan degradation process-related gene thaumatin-like protein (*TLP*) was alternatively spliced in WT strains via retained introns, leading to the production of a longer *TLP1* isoform and a shorter *TLP2* isoform; however, only the *TLP2* isoform was observed in *PRMT5*i strains. Experiments examining the polysaccharide content of the TLP silencing, *TLP1* overexpression (OE-*TLP1*), and *TLP2* overexpression (OE-*TLP2*) transformants revealed that *TLP2* plays a more important role than *TLP1* in polysaccharide degradation. Through a combination of yeast two-hybrid, bimolecular fluorescence complementation and surface plasmon resonance assays, we found that *TLP2* directly physically interacted with phosphoglucose isomerase (PGI), a key enzyme in polysaccharide synthesis, and thereby increased PGI activity. However, TLP1 failed to interact with PGI, and PGI activity was not affected. Further inspection showed that the polysaccharide content was decreased in the OE-*TLP2* strains but not significantly changed in the OE-*TLP1* strains compared with that in the WT strains. In addition, the polysaccharide content of the *PRMT5-TLP*-cosilenced strains was not significantly different from that of the WT strains. These results demonstrate that PRMT5 modulates *TLP* processing of pre-mRNA transcripts and thereby decreases the polysaccharide content.

**IMPORTANCE:**

PRMT5 contributes to secondary metabolite biosynthesis in *Ganoderma lucidum*. However, the mechanism through which PRMT5 regulates the biosynthesis of secondary metabolites remains unclear. In the current study, *PRMT5* silencing led to a significant decrease in the biosynthesis of polysaccharides from *G. lucidum* through the action of the alternative splicing of *TLP*. A shorter *TLP2* isoform can directly bind to PGI and regulated polysaccharide biosynthesis. These results suggest that PRMT5 enhances PGI activity by regulating TLP binding to PGI. The results of the current study reveal a novel target gene for PRMT5-mediated alternative splicing and provide a reference for the identification of PRMT5 regulatory target genes.

## INTRODUCTION

Protein arginine methyltransferase 5 (PRMT5) is the primary type II arginine methyltransferase and the major enzyme responsible for the mono- and symmetric dimethylation of histones and nonhistone substrates. Its histone substrates, including arginine 3 of histone H4 (H4R3), arginine 2 of histone H3, arginine 8 of histone H3, and arginine 3 of histone H2A, are involved in gene expression and protein–protein interactions (1). Its nonhistone substrates include three subunits of the survival of motor neuron complex (SmB, SmD1, and SmD3) that are involved in the assembly of small nuclear ribonucleoproteins (snRNPs), which are essential components of the spliceosome machinery (2). PRMT5 activity is essential in a wide range of biological processes, including cellular growth and differentiation, chromatin regulation, transcript splicing, and cell signaling through histone and nonhistone modification (3). RNA interference-mediated depletion of PRMT5 in planarian stem cells results in a reduced organism size and increases in the transposon and repetitive element transcript levels (4). Mutation of the *Arabidopsis* PRMT5 protein reduces the shoot regeneration rate by decreasing the frequency and capacity of shoot regeneration and the number of shoots per callus (5). In contrast to animals and plants, only a few studies have investigated PRMT5 in fungi. Skb1 (a homolog of mammalian PRMT5) is needed for the normal cell viability and polarity of fission yeast in a hyperosmotic medium (6). Disruption of Hsl7 (a homolog of mammalian PRMT5) increases the cell length and filamentous response under low-ammonium conditions in the plant pathogen *Ustilago maydis* (7). Previous research has also shown that PRMT5 contributes to secondary metabolite pigment biosynthesis in *Penicillium expansum* (8). This area of research has attracted widespread interest. However, the mechanism through which PRMT5 regulates the biosynthesis of secondary metabolites remains unclear.

Mechanistic studies have revealed two major modes of PRMT5 action. The direct induction of histone methylation is involved in multiple physiological processes. For example, the symmetric methylation of histone H4R3 by PRMT5 modulates oligodendrocyte differentiation and developmental myelination (9). PRMT5 also regulates physiological functions through methylation of nonhistone substrates. For example, PRMT5 inhibition alters the methylation status of the E2F1 protein, leading to DNA damage repair attenuation, cell cycle arrest, and increased apoptosis (10). Another physiological function that is regulated by PRMT5 through nonhistone substrate methylation is spliceosome methylation, which is a global requirement for pre-mRNA splicing and is involved in many physiological processes (11). PRMT5 promotes the methylation of Sm spliceosomal proteins and significantly alters the spliced repertoire of mouse double minute four and euchromatic histone lysine N-methyltransferase 2 RNAs in mammalian embryonic cells and primordial cells (12). Moreover, PRMT5 is needed for methylation of the Sm spliceosome and affects EIF4E activity, and the OGT intron retention interface impairs the growth of tumor cells (13). Loss of PRMT5 function-induced chilling-sensitive 3 mRNA splicing results in enhanced resistance of *Arabidopsis thaliana* to the virulent oomycete pathogen *Hyaloperonospora arabidopsidis* (14). *Arabidopsis* PRMT5 mutants regulate shoot regeneration via abnormal pre-mRNA splicing during related to KPC1 and KIP-related protein gene transcription (5). There is a need to identify and verify additional new target genes and to confirm the mechanisms through which PRMT5 regulates secondary metabolite biosynthesis.

Thaumatin-like protein (TLP) belongs to a family of pathogenesis-related proteins that share high sequence homology with thaumatin, an intensely sweet-tasting disulfide protein that was originally identified in the fruit of the shrub *Thaumatococcus daniellii* (15). TLP itself does not have a sweet taste, but as a member of the PR-5 gene family, TLP is associated with a broad range of growth, development, stress responses and host defenses in plants, fungi, and animals (16). The TLP1 gene expression level of medium-size plants possibly contributes to generative organ development in *Corydalis cava* (17). The TLP expression level in broccoli is significantly higher under salt and drought stress conditions, and transgenic plants overexpressing TLP exhibit high salt and drought tolerance (18). TLP not only plays a critical role in the stress response but also is considered an important antimicrobial. Transcriptome sequencing of highly resistant and susceptible tomato (*Solanum lycopersicum*) varieties identified TLP as a candidate gene for resistance against late blight (19). TLP expression is induced during the wheat defense response to leaf rust attack and increases antifungal activity against *Puccinia triticina* (10). Much research on TLP has focused on its role in plants, and relatively few studies have focused specifically on the function of TLP in fungi. The expression of TLPs of Polyporales is upregulated in response to pine and aspen, suggesting that they may be new candidate factors in wood decomposition (20). TLP purified from the *Lentinula edodes* fruiting body exhibits lentinan-degrading activity (21). However, the physiological and biochemical functions of TLPs in fungi are unclear, and this issue requires further research.

*Ganoderma lucidum* has been a traditional medicinal fungus in Asia for thousands of years due to its broad medicinal properties and wide variety of biological activities. *G. lucidum* polysaccharides are the major important active constituents and components used in the medical application of *G. lucidum. G. lucidum* polysaccharides have been studied for several years and have been demonstrated to possess diverse bioactivities, such as anti-tumor, anti-oxidative, anti-microbial, cytotoxic, hepatoprotective, anti-hypertensive, and immunomodulatory activities (22). *G. lucidum* has potential high commercial value for both food and biopharmaceutical and food industry applications. Therefore, most of the current research has mainly focused on optimizing the fermentation conditions for *G. lucidum* polysaccharide biosynthesis and cloning the key genes in the *G. lucidum* polysaccharide biosynthesis pathway. The key enzymes in the *G. lucidum* polysaccharide biosynthetic pathways include phosphoglucose isomerase (PGI), phosphoglucose mutase (PGM), and phosphomannose isomerase (PMI) (23, 24). However, very few studies have investigated the key genes and molecular mechanisms regulating *G. lucidum* polysaccharide biosynthesis. Therefore, it is important to identify new genes and to analyze the possible mechanisms within regulatory networks of *G. lucidum* polysaccharide biosynthesis. Knowledge about the mechanisms present in *G. lucidum* that regulate polysaccharide biosynthesis could also provide a model and foundation for further research on the molecular mechanism of the key genes that regulate secondary metabolite biosynthesis in fungi.

PGI, as the key component in the glycolysis and gluconeogenesis pathways, catalyzes the isomerization of glucose-6-phosphate (G6-P) and fructose-6-phosphate (25). PGI is a multifunctional enzyme that is involved in regulating growth, hyphal polarity, stress resistance, and cell-wall integrity (26). A point mutation in the PGI gene in *Aspergillus nidulans* results in abnormal development by primary germ tube extension, yielding an enlarged, swollen apex with pronounced wall thickening (27). The deletion of PGI significantly inhibits hyphal growth, conidial germination, and deoxynivalenol biosynthesis in *Fusarium graminearum* (28). In addition, PGI is a key enzyme that regulates polysaccharide biosynthesis and metabolism. G6-P is a substrate used by PGM to produce glucose-1P, which is the central precursor in polysaccharide synthesis (29). Therefore, higher PGI activity is associated with increased G6-P flux into the glycolysis pathway. Increased PGI activity results in reduced levels of a major precursor needed for polysaccharide synthesis, G6-P, and an increased lactate yield (30). PGI silencing exerts a significant increase in the extracellular polysaccharide (EPS) and intracellular polysaccharide (IPS) levels in *Lentinula edodes* (31). Significantly lower PGI activity is observed from days 4 to 8 of culture after coixenolide addition than under control conditions, and this decreased activity is associated with increases in the EPS and IPS content in *G. lucidum* (32). PGI activity decreases over time during fermentation with glucose as the carbon source, but significant increases in the EPS and IPS contents of *G. lucidum* are observed (23).

In this study, we aimed to understand the mechanism through which PRMT5 contributes to polysaccharide biosynthesis. We found that target gene (*TLP*) splicing regulated by the silencing of *PRMT5* led to a decrease in the polysaccharide content of the fungus. The results identified an uncharacterized mechanism through which PRMT5 regulates secondary metabolite biosynthesis and revealed a new mechanism through which TLP decreases polysaccharide biosynthesis.

## RESULTS

### The silencing of *PRMT5* decreases the polysaccharide content of *G. lucidum*

**Fig 1:**
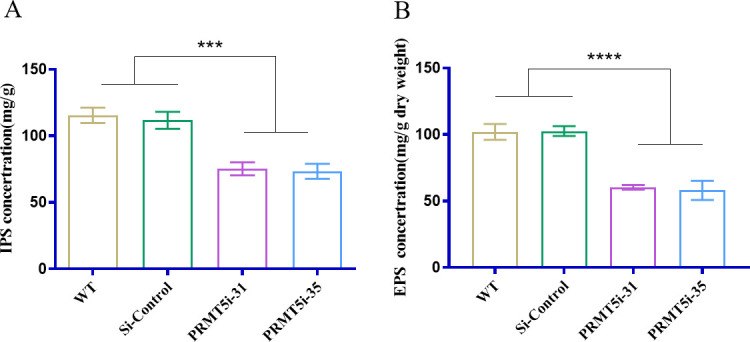
Polysaccharide content of *G. lucidum* WT and PRMT5-silenced strains. (A) IPS content of WT and *PRMT5*i strains of *G. lucidum*. (B) EPS content of the WT and *PRMT5*i strains. The data are presented as the means ± standard deviations of the values obtained from three independent experiments. ***P < 0.001, ****P < 0.0001 by one-way analysis of variance).

To understand the links connecting PRMT5 with polysaccharide biosynthesis in *G. lucidum*, *G. lucidum* strains in which the *PRMT5* gene (GenBank: OP360010, the silenced fragments in the conserved *PRMT5* oligomerization domain) was silenced were constructed by RNA interference (RNAi) using cassette plasmids produced by our laboratory (33). Hygromycin-resistant transformants were isolated, and *PRMT5* gene knockdown was confirmed by real-time quantitative reverse transcription PCR (qRT–PCR). The *PRMT5*-silenced strains (*PRMT5*i-31 and *PRMT5*i-35) produced in this study exhibited 68% and 72% reductions in the *PRMT5* gene expression level compared with that of the wild-type (WT, ACCC53264) and Si-control (empty vector control) strains (Fig. S1A). The expression of the PRMT5 protein was confirmed by Western blot analysis and also showed that PRMT5 silencing reduced efficiently the PRMT5 protein level (Fig. S1B). qRT‒PCR and Western blot results confirmed the efficiency of *PRMT5* silencing in the *PRMT5*-silenced strains. Polysaccharides and triterpenes are considered the main bioactive components in *G. lucidum* (34). Therefore, the polysaccharide content of the WT and *PRMT5*i strains was measured. The silencing of *PRMT5* led to a significant decrease in the IPS and EPS contents (Fig. 1A). The IPS and EPS contents of the *PRMT5*i strains were decreased by 35% and 41% compared with those of the WT and Si-control strains, respectively (Fig. 1B). These data suggest that the silencing of *PRMT5* decreases the polysaccharide content of *G. lucidum*, including that of IPS and EPS.

### *PRMT5* silencing reduces symmetric arginine methylation of the spliceosome factor SmD3

**Fig 2:**
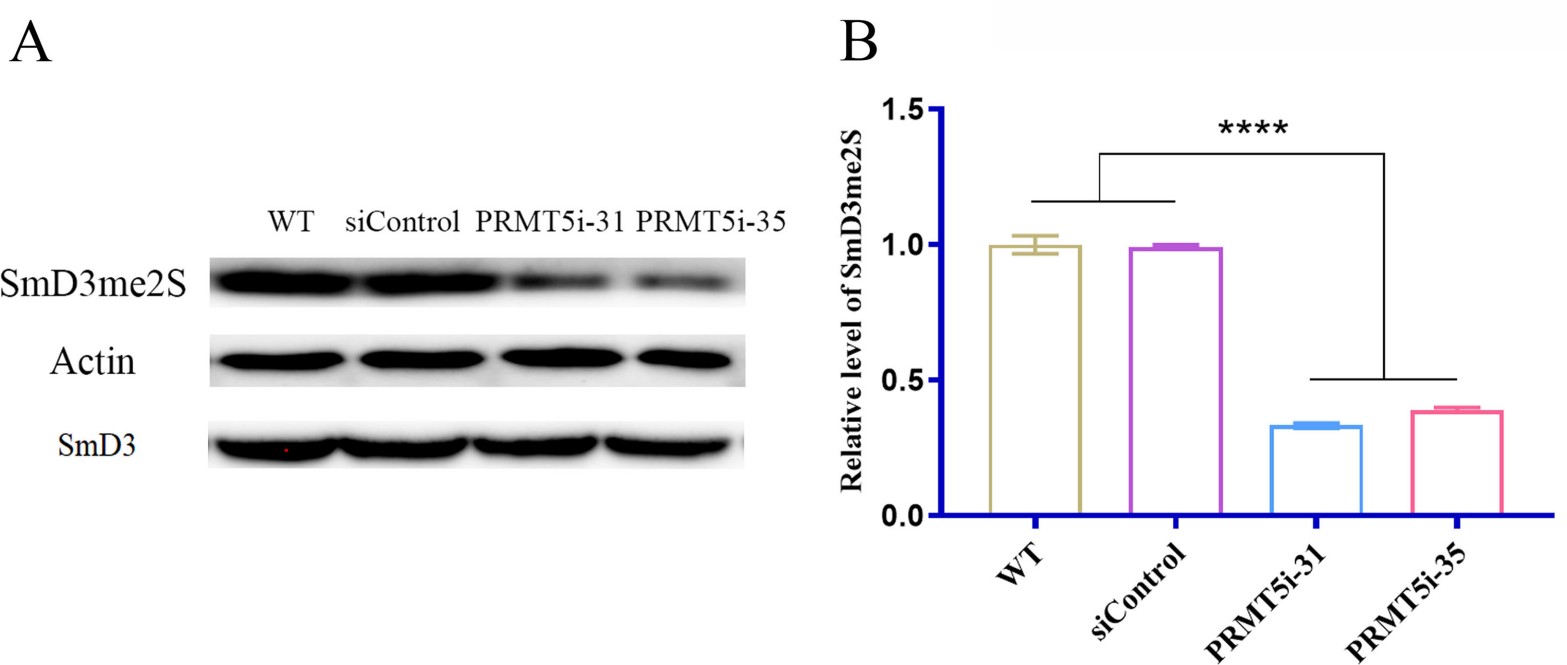
*PRMT5* silencing decreases the SmD3me2S levels of *G. lucidum*. (A) SmD3me2S levels of the WT and *PRMT5*i strains were confirmed by Western blot analysis. The anti-SYM11 antibody specifically recognizes symmetric dimethylated arginine-containing peptides in various species. A band at 16 kDa corresponds to symmetrically demethylated SmD3 proteins; β-actin and SmD3 were used as internal reference for data normalization. (B) Grayscale analysis of SmD3me2S levels of the WT and *PRMT5*i strains. The data are presented as the means ± standard deviations of the values obtained from three independent experiments. ****P < 0.0001 by one-way ANOVA.

PRMT5 methylates proteins involved in RNA splicing, one of which is SmD3 (35, 36). Thus, to identify the PRMT5 that catalyzes SmD3 methylation, the SmD3 methylation levels in WT *G. lucidum* strains and in *PRMT5*i-31 and *PRMT5*i-35 strains were measured by Western blotting with antibodies against SYM11 (37). The anti-SYM11 antibody specifically recognizes symmetric dimethylated arginine-containing peptides in various species (35, 38). This antibody recognizes a band at 16 kDa corresponding to SmD3 and proteins that contain arginine that are symmetrically demethylated. β-Actin was used as an internal reference for data normalization. The results showed that the PRMT5-catalyzed methylation of arginine residues in SmD3 was significantly reduced in the *PRMT5*-silenced strains compared with that in the WT and Si-control strains and did not affect the expression level of the Smd3 protein (Fig. 2A). The signal produced by the binding of the antibody to the methylated protein was quantified using image analysis software, and the results indicated that SmD3 methylation was approximately 60% lower in the *PRMT5*i strain than in the WT strain (Fig. 2B). These results suggest that PRMT5 activity is reduced in *PRMT5*-silenced strains and that the silencing of PRMT5 decreases SmD3 methylation.

### *PRMT5* silencing leads to splicing changes in *G. lucidum*

**Fig 3:**
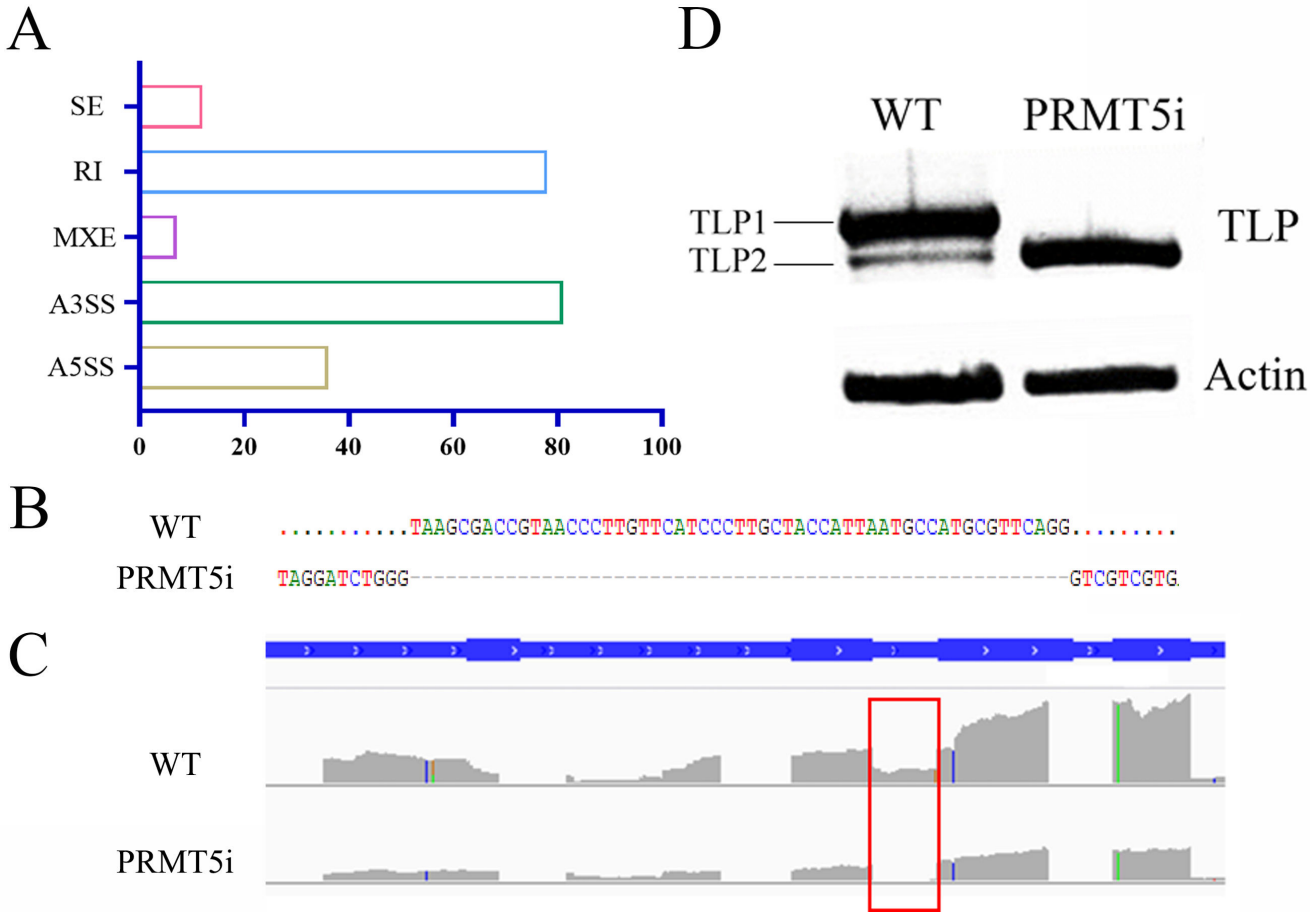
Differentially spliced genes in the *PRMT5*-silenced and WT strains. (A) The number of alternative splicing events in the PRMT5-silenced strains vs WT strains was determined by RNA-seq analysis. Cassette exons (SEs), retained introns (RIs), mutually exclusive exons (MXEs), alternative 5′ splice sites (A5SSs) and alternative 3′ splicing sites (A3SSs). (B and C) Pre-RNA splicing of TLP in the WT and *PRMT5*i strains. (D) Semiquantitative PCR of the indicated transcripts in the WT and PRMT5i strains.

PRMT5-regulated SmD3 methylation is a requirement for pre-mRNA splicing, and PRMT5 activity in the control of pre-mRNA splicing is conserved (39). To understand the effect of *PRMT5* silencing on alternative splicing and to determine whether PRMT5 regulates polysaccharide biosynthesis in *G. lucidum* through gene splicing, we performed an RNA-seq analysis of *PRMT5*i and WT strains (the raw sequence reads are deposited in the National Center for Biotechnology Information Sequence Read Archive, accession number PRJNA933587). Replicate multivariate analysis of transcript splicing, an unbiased approach, was used to determine how *PRMT5* silencing affects RNA splicing in *G. lucidum*. Using this approach, we identified a total of 214 differential alternative splicing events that were affected by *PRMT5* silencing. These splicing events were associated with 185 unique genes. The largest proportion of novel alternatively spliced transcripts was generated by retained introns (RIs) and alternative 3′ splicing sites (Fig. 3A). Because the presence of abnormal introns retained/loss is an important method for measuring PRMT5 function (40), we screened 78 spliced RI genes. A Kyoto Encyclopedia of Genes and Genomes (KEGG) pathway analysis of these genes showed that abnormally spliced RI genes are implicated in metabolism. TLP is known to play a vital role in glycan degradation. *TLP* in WT strains was found to undergo alternative splicing at intron 4, and intron 4 was lost in the *PRMT5*i strains (Fig. 3B and C). Hence, *PRMT5* silencing leads to loss of introns in the TLP transcript. These findings were confirmed by semiquantitative PCR amplification of a fragment encompassing exons 3–5. We observed that the WT strains produced two alternative transcripts, a longer isoform (*TLP1*) and a shorter isoform (*TLP2*). The longer *TLP* isoform was not observed in the *PRMT5*i strain (Fig. 3D), suggesting that PRMT5 might be essential for the regulation of splicing during *TLP* expression. The alternative splicing of *TLP* also results in changes in its conserved domains, and it is speculated that gene function is altered by these changes.

### *G. lucidum* PRMT5 regulates polysaccharide biosynthesis through *TLP* alternative splicing

**Fig 4:**
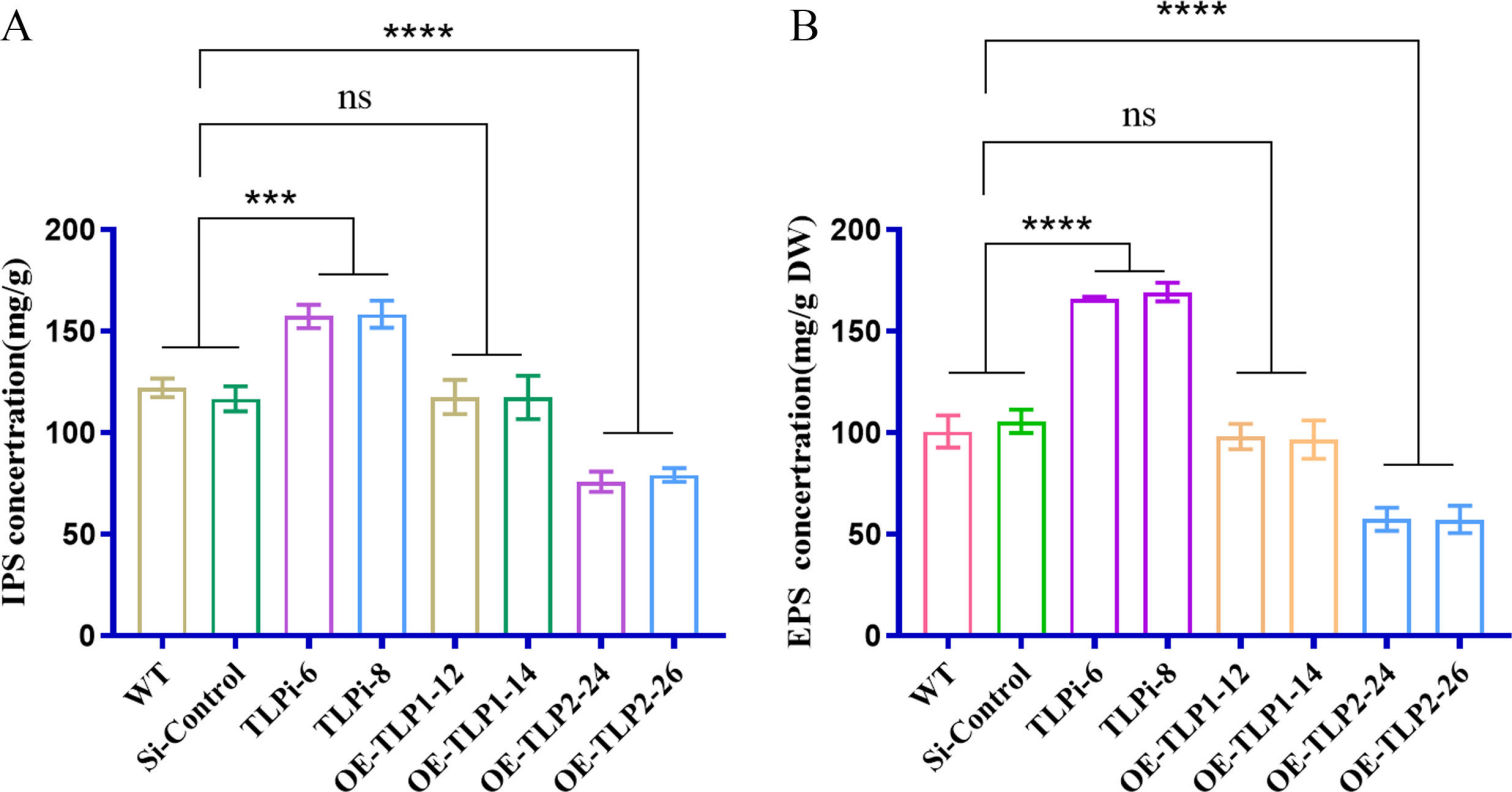
Polysaccharide contents of the *G. lucidum* WT, *TLP*i, OE-*TLP1*, and OE-*TLP2* strains. (A) IPS contents of the WT, *TLP*i, OE-*TLP1*, and OE-*TLP2* strains of *G. lucidum*. (B) EPS contents of the WT, *TLP*i, OE-*TLP1*, and OE-*TLP2* strains. The data are presented as the means ± standard deviations of the values obtained from three independent experiments. ***P < 0.001, ****P < 0.0001 by one-way ANOVA. NS, not significant.

To determine whether polysaccharide biosynthesis is affected by alternative splicing of *TLP*s, *TLP*-silenced and isoform-overexpressing transformants were constructed. The efficiency of TLP silencing and overexpressing was confirmed by qRT–PCR (Fig. S2A through C). The two TLP-silenced strains were named polysaccharide content of the TLP silencing (TLPi)-6 and TLPi-8; the longer TLP isoform-overexpressing strains were named *TLP1* overexpression (OE-TLP1)-12 and OE-TLP1-14, and the shorter TLP isoform-overexpressing strains were named *TLP2* overexpression (OE-TLP2)-24 and OE-TLP2-26). The expression levels of the *TLP1* and *TLP2* genes were determined by Northern blot analysis using two specific probes for detecting *TLP1* and *TLP2*, respectively. Northern blot analysis showed that TLP1 and TLP2 silencing reduced efficiently the TLP expression level (Fig. S2D through E). The expression of the *TLP2* gene was significantly increased in the OE-*TLP2* strains, as detected with a *TLP2*-specific probe (Fig. S2E). qRT‒PCR and Northern blot results confirmed the efficiency of *TLP1* and *TLP2* gene expression in the *TLP1* and *TLP2* strains, respectively. The IPS and EPS contents of *TLP*-silenced strains and OE-*TLP1/TLP2* strains were examined. The results showed that the IPS and EPS contents were significantly increased by 65% and 32% in the *TLP*-silenced strains and significantly decreased by 43% and 35% in the OE-*TLP2* strain compared with those in the WT strain. However, the OE-*TLP1* strain showed no significant difference in the polysaccharide content compared with that in the WT strain (Fig. 4). These results show that the shorter *TLP* isoform *TLP2* inhibits total polysaccharide biosynthesis and that *TLP1* does not affect polysaccharide biosynthesis. The results suggest that *TLP2* plays a more important role than *TLP1* in controlling the polysaccharide content of *G. lucidum* and that PRMT5 regulates polysaccharide biosynthesis in *G. lucidum* through *TLP* mRNA splicing.

**Fig 5:**
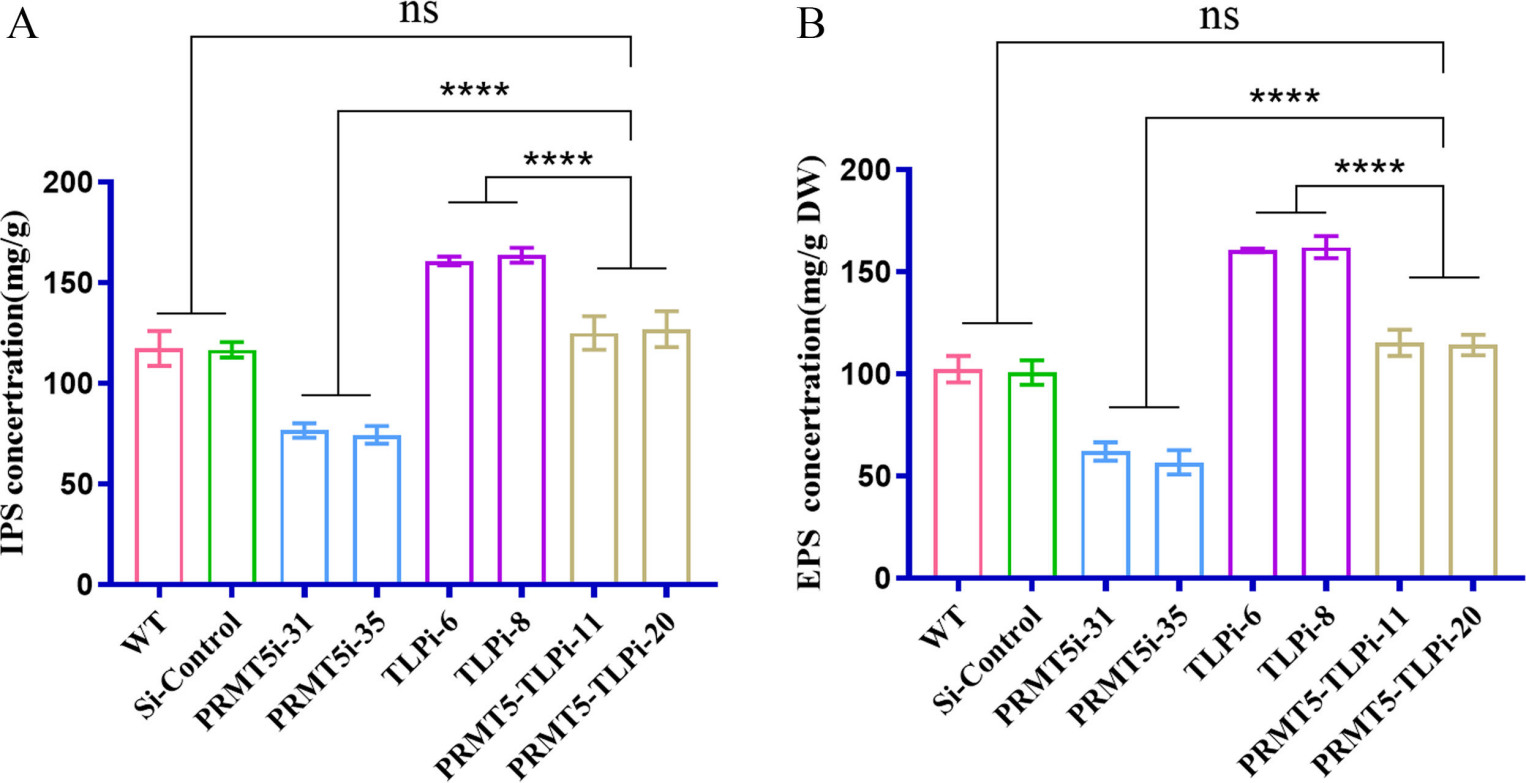
Polysaccharide contents of the *G. lucidum* WT, *PRMT5*i, *TLP*i, and *PRMT5–TLP*i strains. (A) IPS contents of the WT, *PRMT5*i, *TLP*i, and *PRMT5–TLP*i strains of *G. lucidum*. (B) EPS contents of the WT, *PRMT5*i, *TLP*i and *PRMT5–TLP*i strains. The data are presented as the means ± standard deviations of the values obtained from three independent experiments. ****P < 0.0001 by one-way ANOVA.

*PRMT5*- and *TLP*-cosilenced strains were constructed and used to illustrate the role of TLP in PRMT5-regulated *G. lucidum* polysaccharide biosynthesis. The construction of *PRMT5–TLP*i-11 and *PRMT5–TLP*i-20 was confirmed by qRT–PCR, and these strains were used for further analysis (Fig. S3; the PRMT5- and TLP-cosilenced strains were named PRMT5–TLPi-11 and PRMT5–TLPi-20). PRMT5 protein expression was confirmed by Western blot analysis with the anti-PRMT5 antibody. β-Actin was used as an internal reference for data normalization. PRMT5 protein expression was significantly reduced in the *PRMT5*- and *TLP*-cosilenced strains compared with that in the WT and Si-control strains (Fig. S1B). qRT‒PCR and Western blot results confirmed the efficiency of *PRMT5* silencing in the *PRMT5*- and *TLP*-cosilenced strains. The expression levels of the *TLP1* and *TLP2* genes were determined by Northern blot analysis using two specific probes for detecting *TLP1* and *TLP2*, respectively. The results showed that the expression of the *TLP1* and *TLP2* genes was significantly reduced in the *TLP*- and *PRMT5*-cosilenced strains compared with that in the WT and siControl strains (Fig. S2D through E). qRT‒PCR and Northern blot results confirmed the efficiency of *TLP1* and *TLP2* gene expression in the *PRMT5*- and *TLP*-cosilenced strains, respectively. The IPS and EPS contents were detected in the *PRMT5*i, *TLP*i, and *PRMT5–TLP*i strains. Although the *PRMT5–TLP*i strain showed a significant decrease in the EPS and IPS contents compared with those of the *TLP*i strain and a significant increase compared with those of the *PRMT5*i strain, no significant difference in the EPS or IPS content was found between the WT strain and *PRMT5–TLP*i strain (Fig. 5). The results show that PRMT5 regulates polysaccharide biosynthesis in *G. lucidum* through *TLP*-mediated alternative splicing events.

### Aberrant splicing of *TLP* affects its interaction with phosphoglucose isomerase

**Fig 6:**
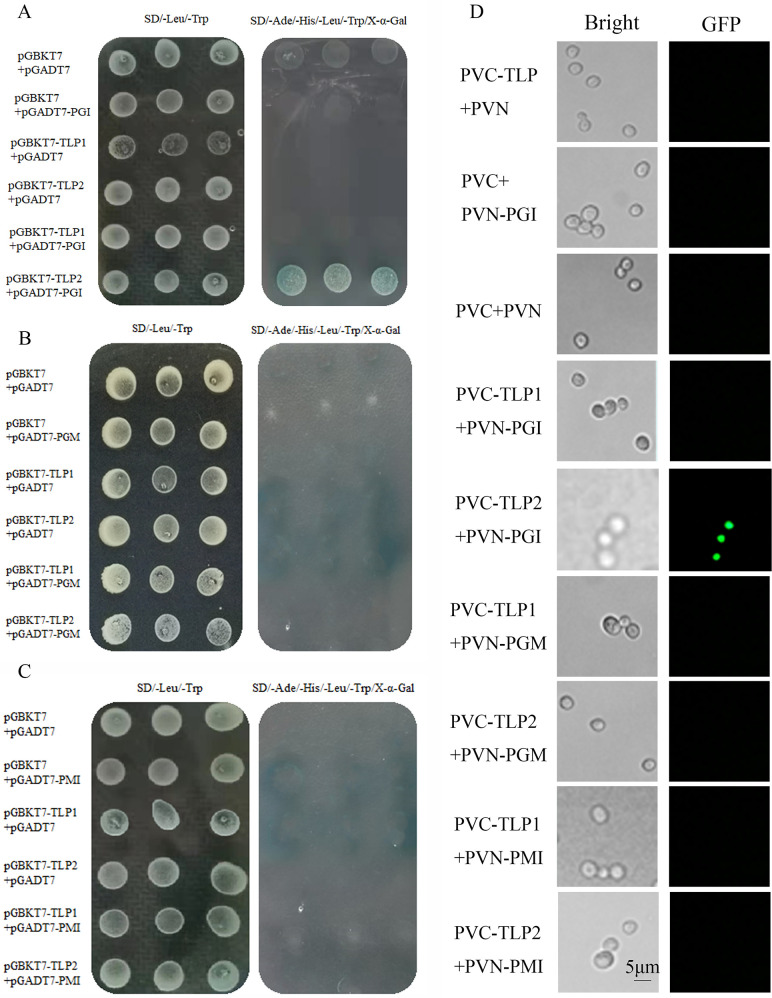
Protein interactions between TLP1/TLP2 and PGI, PGM, or PMI tested by yeast two-hybrid (Y2H) and bimolecular fluorescence complementation (BiFC) assays. (A) Y2H assays showing that TLP2 interacts with PGI. No direct interaction between TLP1 and PGI was detected. (B) Y2H assays showing no direct interaction between TLP1 or TLP2 and PGM. (C) Y2H assays showing no direct interaction between TLP1 or TLP2 and PMI. (D) BiFC-identified interactions of TLP1/TLP2 with PGI, PGM, and PMI. The BiFC signal was observed only for TLP1 and PGI. The interaction between TLP and PGM/PGI/PMI was detected using a Y2H assay. pGBKT7 + pGADT7, pGBKT7 + pGADT7-PGI, and pGBKT7-TLP + pGADT7 were used as controls, and pGBKT7-53 and pGADT7-T were used as controls. pGADT7-PGI/PMI/PGM was used as the bait protein, and pGBKT7-TLP was used as the prey protein. The BiFC assay indicated a correlation between PGM/PMI/PGI and TLP. Fluorescence was detected by constructing the PVN-PGI/PGM/PMI vector and a PVC-TLP vector containing a GFP tag and cotransforming yeast cells with these vectors. Digital image correlation (DIC) images indicate the yeast cell morphology under the normal white-field view; Venus shows the morphology of the yeast cells under green fluorescence. Scale bar: 5 µm.

To understand the molecular mechanism through which the PRMT5-regulated *TLP* alternative splicing event modulates polysaccharide biosynthesis in *G. lucidum*, the relationship between TLP and the key enzymes of polysaccharide synthesis and metabolism was investigated. Yeast two-hybrid assays were conducted to identify the various interactions that occur among the two TLP isoforms and PGI, PGM, and PMI. As shown in Fig. 6A through C, TLP2 interacts physically with PGI, whereas no direct interaction between TLP2 and PGM or PMI was detected. Furthermore, TLP1 does not bind PGM or PMI and does not interact with PGI (Fig. 6A through C).

**Fig 7:**
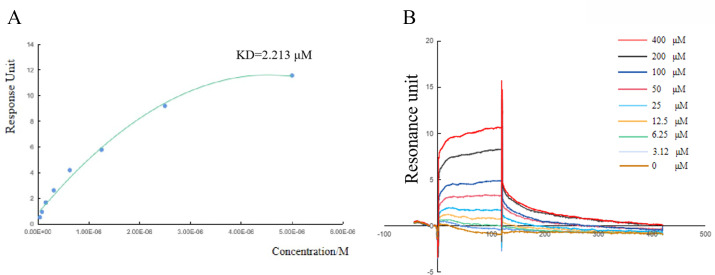
Binding affinity between TLP2 and PGI proteins investigated by surface plasmon resonance assays. (A) KD values of TLP2 and PGI proteins. (B) Binding curves for the interaction of TLP2 and PGI proteins. PGI was loaded onto a chip, and the TLP2 protein was injected as the analyte at concentrations of 0–400 nM. Different colored lines represent different concentrations of the analytes.

To further verify the interaction between TLP1/TLP2 and PGI, PGM, or PMI, bimolecular fluorescence complementation (BiFC) assays were conducted. The results demonstrated that TLP2 interacts physically and directly with PGI but has no direct interaction with PGM or PMI (Fig. 6D). Because TLP1 failed to interact with any of the three proteins, yeast two-hybrid and BiFC assays together showed that TLP2 interacts only with PGI. To further determine how TLP2 interacts with the PGI protein, a surface plasmon resonance (SPR) assay was performed. The experiments revealed strong binding affinity between PGI and TLP2, with a KD value of 2.213 µM (Fig. 7A). The exposure of immobilized PGI to increasing concentrations (0–400 μM) of TLP2 protein resulted in the detection of increased binding (Fig. 7B). These results indicate that TLP2 can directly bind to PGI and that PRMT5 regulates the interaction between TLP and PGI through TLP splicing.

### Aberrant *TLP* splicing affects its binding to PGI and thereby regulates enzyme activity

**Fig 8:**
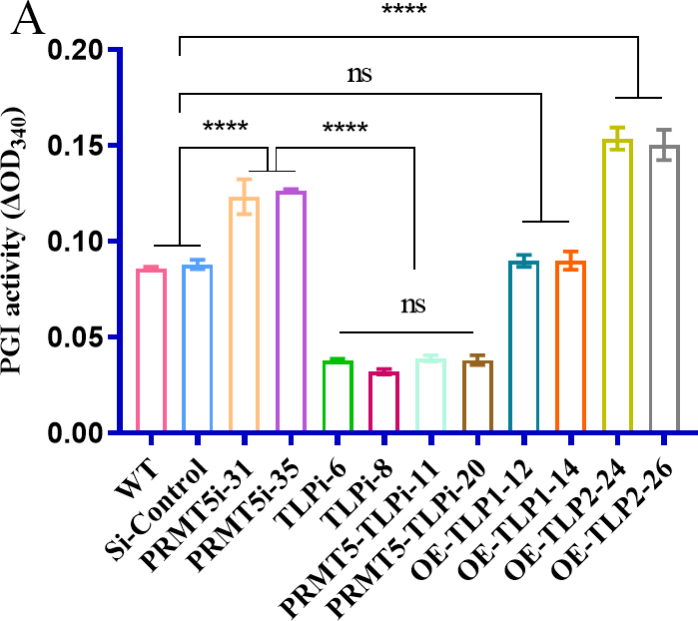
PGI activity of *G. lucidum*. (A) PGI activity of the WT, *PRMT5*i, *TLP*i, *PRMT5–TLP*i, and OE-*TLP1/TLP2* strains. The data are presented as the means ± standard deviations of the values obtained from three independent experiments. ****P < 0.0001 by one-way ANOVA.

Because enzyme activity is often regulated by protein–protein interactions, PGI activity in *G. lucidum* was measured to understand the effect of the interaction of TLP with PGI. PGI activity was significantly higher (66%) in the OE-*TLP2* strains than in the WT strain, and no significant difference in PGI activity was observed between the OE-*TLP1* and WT strains. In addition, PGI activity was increased by 45% in the *PRMT5*-silenced strains compared with that in the WT strain. A significant 53% reduction was detected in the *TLP*-silenced strains compared with that in the WT strains, but no significant change was found in the *TLP*i strains or the *PRMT5*- and *TLP*-silenced strains (Fig. 8). These results show that PRMT5 enhances PGI activity through TLP. Furthermore, these results suggest that PRMT5 enhances PGI activity by regulating the binding of TLP to PGI.

## DISCUSSION

**Fig 9:**
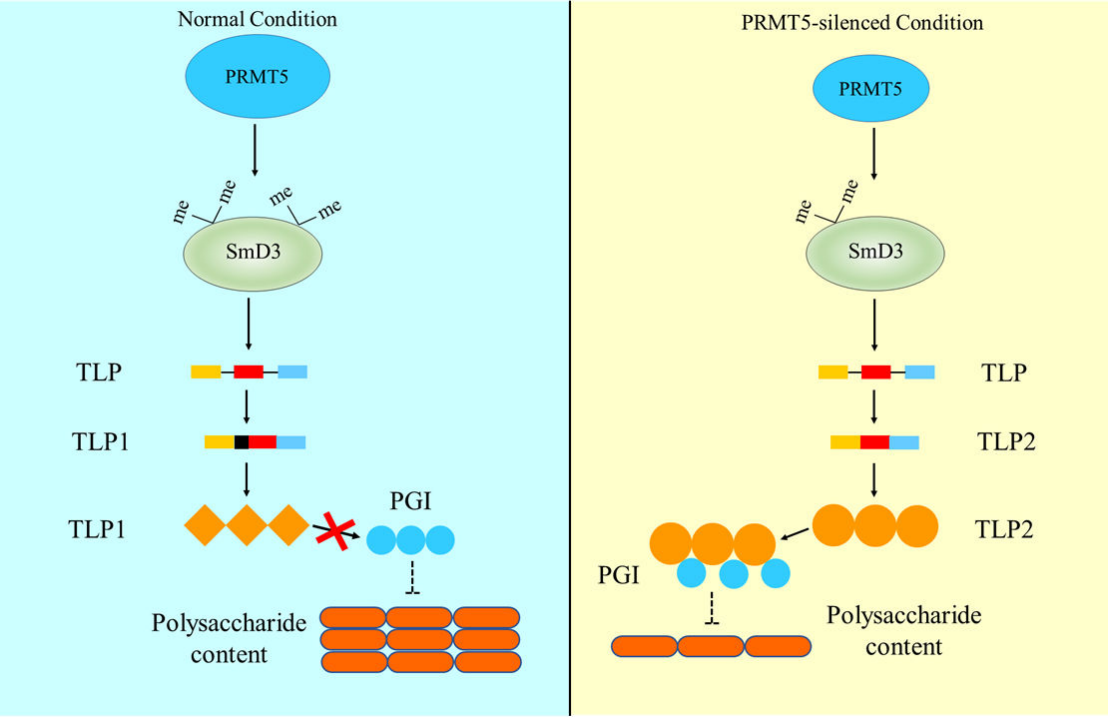
Schematic describing a hypothetical model of the mechanism by which PRMT5 mediates polysaccharide biosynthesis in *G. lucidum* by controlling *TLP* splicing. *PRMT5* silencing decreased the polysaccharide biosynthesis in *G. lucidum* by splicing *TLP* pre-mRNA to *TLP2*. TLP2 plays a more important role than TLP1 in polysaccharide biosynthesis in *G. lucidum*. TLP2 directly physically interacts with PGI and increases PGI activity, thereby decreasing the polysaccharide content. TLP1 failed to interact with PGI, and the polysaccharide content of the cells was not affected. In summary, PRMT5 modulates *TLP* pre-mRNA transcript processing, thus regulating polysaccharide biosynthesis. The solid black arrows in the hypothetical model indicate steps supported by data from experiments performed in the present study; the dotted arrows indicate steps supported by data derived from other systems.

PRMT5 is a type II methyltransferase that catalyzes the transfer of a methyl group from S-adenosylmethionine to arginine residues of histone and nonhistone proteins (35). Among these modifications, the methylation of nonhistone proteins, especially the spliceosome Sm protein, modulates constitutive and alternative pre-mRNA splicing of diverse genes and is an important way in which PRMT5 performs its functions (36). PRMT5 has been shown to participate in various cellular processes, including transcriptional regulation, signal transduction, and stress responses, by mediating alternative pre-mRNA splicing (41, 42). For example, in human acute myeloid leukemia cells, loss of PRMT5 leads to changes in the alternative splicing of multiple essential genes that regulate cell survival, including PNKP and PDCD2 (43). Mutations in PRMT5 alter alternative splicing of the core-clock gene PSEUDO RESPONSE REGULATOR 9 and contribute to regulation of the circadian rhythm in *Arabidopsis thaliana* (44). A change in PRMT5 activity modulates transcription of the stress-related gene *At*1G18160 and eventually boosts tolerance to salt stress in *Arabidopsis* (45). Mutation of PRMT5 alters the methylation of snRNP Sm and influences pre-mRNA splicing, conferring high salt tolerance in *Arabidopsis* (38). The knockdown of PRMT5 results in an increase in the frequency of I-6 splicing of the circadian clock gene in *Neurospora* (46). PRMT5 modulates diverse phenotypes by regulating the splicing of a variety of target genes; thus, identifying new target mRNA splicing genes is key to understanding the mechanism of action of PRMT5. In the current study, *PRMT5* silencing led to a significant decrease in the biosynthesis of polysaccharides from *G. lucidum* through the alternative splicing of *TLP* (Fig. 9). These results are a common example of the various physiological functions of the alternative gene-splicing events that are regulated by PRMT5. The results of the current study reveal a novel target gene of PRMT5-mediated alternative splicing and provide a reference for the identification of regulatory target genes of *PRMT5*.

TLPs are the products of a large, highly complex gene family, the members of which are associated with a broad range of host defense and stress response processes (16). Although TLPs share similar structures within their families, their functions show some differences. Recently, TLPs have been discovered in a wide range of plants, and their defense and stress response activities have been explored (47). However, few studies have studied the TLP-mediated regulation of physiological functions in fungi. The *cetA* (homology to plant TLP) mutant in *Aspergillus nidulans*, which contains a mutation in this glucose-repressible gene, exhibits delayed germination, abnormal hyphal branching, and cell-wall defects (48). Deletion of the TLPs *cetA* and *calA* in *Aspergillus nidulans* yields a synthetic lethal phenotype, and a few cells show cell-wall defects and lysis during germination and abnormal hyphal branching (49). The current research shows that TLP2 regulates polysaccharide biosynthesis in *G. lucidum* and that the *TLP* gene transcript undergoes alternative splicing. To the best of our knowledge, this study provides the first description of the mechanism through which TLP2 contributes to secondary metabolite biosynthesis via alternative splicing. Although TLP is known to be important for physiological processing, the functional mechanism through which it acts in physiological processing remains unclear. Barley TLP8 binds to insoluble (1,1,3,4)-β-D glucans in grain extracts and thereby facilitates the removal of this polysaccharide during the final beer-making process (50). TLP, as a putative SGC interactor, suppresses callose deposition in the nascent generative cell by digesting β-1,3-glucans, the main constituent of callose in *Arabidopsis* (51). However, our understanding of the mode of action of TLP in fungi is incomplete, and the subject remains under debate. The current study found that TLP2 regulated *G. lucidum* polysaccharide biosynthesis by interacting with PGI, a key enzyme in polysaccharide metabolism, and induced an increase in its activity without a direct interaction with PGM. We speculated that TLP2 does not interact with PGM, which may be due to the lack of a binding site in the protein or an effect of the protein spatial structure. TLP2 interacts with PGI and thus increases its activity, which may be due to an influence on other posttranslational modifications or protein stability. The relevant mechanisms have also been observed in other studies (52). PGI is a key enzyme that regulates polysaccharide biosynthesis and metabolism. Changes in PGI activity may directly affect polysaccharide biosynthesis. Therefore, TLP2 regulates polysaccharide biosynthesis through increases PGI activity. This study elucidated the molecular mechanism by which TLP2 regulates physiological function in fungi. Specifically, our findings elucidated the mechanism through which TLP2 contributes to secondary metabolite biosynthesis and may provide a basis for further functional investigation of these gene families.

In the present study, increased PGI activity led to a decreased polysaccharide content in *G. lucidum*. The results of this study are consistent with other recent reports and suggest that PGI is a key enzyme for polysaccharide biosynthesis. Although we found that PGI regulates polysaccharide biosynthesis, the influence of the PGI gene on the physiological functions of polysaccharide biosynthesis is currently unknown. The PGI gene deletion mutant exhibited higher total cellulase activity and pellet-like growth, and a possible mechanism could be the availability of building block precursors produced by glycolysis and the pentose phosphate pathway (26). In the present study, only the interaction of TLP2 with PGI was found to decrease the polysaccharide content. We speculated that TLP1 does not interact with PGI due to the lack of a binding site residue in the protein or the effect of the protein spatial structure. The alternative splicing of TLP1 also results in changes in its conserved domains, and it is speculated that gene function is altered by these changes. Whether there are other mechanisms that regulate polysaccharide biosynthesis in *G. lucidum* remains unknown. These issues will be investigated in future work.

## MATERIALS AND METHODS

### Strains and growth conditions

In this study, *G. lucidum* ACCC53264 provided by the Agricultural Culture Collection of China was used as the WT strain. The WT and transformant strains generated in this study were maintained on potato dextrose agar (PDA) slants. A 0.5-cm^3^ inoculation block was obtained and inoculated into complete yeast medium (CYM) plates. The mycelia were grown on complete medium (CYM, 2% glucose, 1% maltose, 0.46% KH_2_PO_4_, 0.2% yeast extract, 0.2% tryptone, and 0.05% MgSO_4_·7H_2_O) for 7 days at 28°C prior to evaluation of the IPS and EPS contents.

### Construction of RNAi strains

The sequences of the *PRMT5* and *TLP* genes in *G. lucidum* were obtained from the database of the *G. lucidum* genome. The coding regions of the *PRMT5* gene and the *TLP* gene were amplified using *G. lucidum* cDNA as the template, and PCR was performed using the primers *PRMT5*i-F (ACTGGGTACCGCTGGAAAGCCCATTACG), *PRMT5*i-R (ACTGACTAGTGACGGAGACATCTGCGAC), *TLP*i-F (ACTGGGTACCCTCGTGGAAGACCAGATT), and *TLP*i-R (ACTGACTAGTGAGTCAAGGCAGCAAAA) to construct the fungal RNAi vectors (53). The RNAi silencing vectors pAN7-dual-*PRMT5*i and pAN7-dual-*TLP*i were electroporated into *G. lucidum* (54). The two independent strains with the highest silencing efficiencies were selected for use in follow-up experiments. The empty vector control was also selected according to the above-described method and named Si-control.

### Estimation of the EPS and IPS concentrations

After the removal of mycelia by centrifugation, crude EPSs were precipitated by adding four volumes of 95% (vol/vol) ethanol, incubating the solution overnight at 4°C, and separating the components by centrifugation. The insoluble components were suspended in 1-M NaOH at 60°C for 1 h, and the supernatants were analyzed using the phenol–sulfuric acid method. The IPS content in the mycelia was measured after drying at 60°C to a constant dry weight; 1-M NaOH was used to extract IPSs at 60°C for 1 h. The supernatant was collected by centrifugation, and the IPS content was measured using the phenol‒sulfuric acid method (55, 56).

### RNA extraction procedure

The WT and *PRMT5*i strains were grown on CYM medium for 7 days at 28°C; 200-mg mycelia frozen in liquid nitrogen were ground to a fine powder using a mortar and pestle; and total RNA was extracted with 1-mL RNAiso Plus (Takara, China). Chloroform (0.2 mL) was added to the solution and well mixed until the solution became milky. The samples were centrifuged at 12,000 *g* for 15 minutes at 4°C after kept at room temperature for 5 minutes. The supernatant was transferred to a new tube and extracted with 0.5-mL isopropanol with gentle shaking for about 10 minutes and was centrifuged at 12,000 *g* for 10 minutes at 4°C. The supernatant was again transferred to a 1.5-mL tube, and a 2.5-fold volume of absolute ethanol was added and mixed thoroughly for precipitating the total RNA at −20°C. Subsequently, the RNA was pelleted at 12,000 *g* for 30 mintes at 4°C, washed in 75% ethanol twice, dried in a vacuum, and re-dissolved in RNase-free water. RNA samples were denatured at suitable temperature to open their secondary structure, and mRNA was enriched by oligo (dT)-attached magnetic beads. Synthesis of the first-strand cDNA and the second-strand cDNA was performed after the RNAs were fragmented. The sequencing libraries were constructed by amplifying the selected fragments by PCR after the end repair process and ligation of adapters.

### RNA-seq analysis

The RNA integrity was assessed using the RNA Nano 6000 Assay Kit and the Bioanalyzer 2100 system (Agilent Technologies, Santa Clara, CA, USA). RNA-seq was performed as described previously (57). The sequencing data were filtered with SOAPnuke; afterward, clean reads were obtained and stored in FASTQ format. The clean data were mapped to the reference genome by HISAT (v.2.1.0). Bowtie2 was applied to align the clean reads to the gene set, in which known and novel, coding, and noncoding transcripts were included.

### Semiquantitative RT–PCR

*G. lucidum* mycelia cultured on PDA were used for semiquantitative RT–PCR detection. The splicing variant-specific primers RT-*TLP*-F (ATGCCGTCGTATC) and RT-*TLP*-R (TCACGACGACC) were used to amplify (28 cycles) two variants of *TLP* with predicted sizes of 306 and 251 bp. Using 18S rRNA as the internal reference gene, the amount of template was determined, and the results were analyzed by gel electrophoresis.

### Immunoblot analysis

Seven-day-old mycelia frozen in liquid nitrogen were ground to a fine powder using a mortar and pestle. The powder was placed in ice-cold extraction buffer. The lysate was centrifuged at 12,000 *g* for 10 minutes at 4°C. Total protein was extracted, separated by 12% SDS-PAGE and transferred to polyvinylidene difluoride membranes (Bio-Rad, USA). Protein gel blot analysis using an antibody against symmetrically dimethylated arginine residues (SYM11;07–413, Millipore) to detect the symmetric arginine methylation of SmD3 revealed a band at 16 kDa (58). Protein gel blotting was used in the immunoblot analysis with a PRMT5 antibody (AB_2615010, Active Motif) to detect the expression level of the PRMT5 protein in the silenced strains. A β-actin-specific antibody (AT0097, CMCTAG) served as the internal control. Blots were developed using the ECL Western blotting detection system (Amersham Bioscience, Sweden). Images were acquired with the Bio-Rad ChemiDoc Touch imaging system (Bio-Rad), and densitometry was performed using ImageJ v.1.8.0 software.

### Yeast two-hybrid assay

Using *G. lucidum* cDNA as a template, we performed PCR amplification of TLP1 and TLP2 fragments. The PCR product was ligated into the pGBKT7 vector to generate the bait vector. The obtained vector was transformed into the yeast Y2HGold strain for the assessment of toxicity and autotranscription activity. The coding sequences (CDSs) of the PMI (GenBank: UOF75523), PGM (GenBank: UOF75521), and PGI (GenBank: UOF75525) genes were then further ligated into the pGADT7 vector to generate feed plasmids that were inserted into strain Y187 via transformation. The yeast two-hybrid strain was obtained by mating the Y187 and Y2HGold strains. The mating cells were screened on synthetic dropout DDO plates (SD-Trp/SD-Leu), and the yeast two-hybrid interactions were assessed on QDO plates (SD-Trp/SD-Leu/SD-Ade/SD-His/X-α-Gal). Protein interactions were assessed based on the expression of different reporter genes under the control of Gal4-responsive promoters.

### Bimolecular fluorescence complementation assay

For the BiFC assay, the PGI, PGM, PMI, TLP1, and TLP2 coding sequences were cloned into pVN and pVC vectors fused with the N‐terminal or C‐terminal sequence to generate pVN-PGI/PGM/PMI and pVC-TLP1/TLP2, respectively. Yeast cells were transfected with the plasmids and incubated in the dark at 30°C; the cells were then imaged using an epifluorescence microscope. DIC images indicate the yeast cell morphology under the normal white-field view; Venus shows the morphology of the yeast cells under green fluorescence.

### Expression and purification of recombinant proteins

The pColdI-TLP2 expression vectors were transformed into *Escherichia coli* strain BL21 (DE3), and protein expression was induced by 0.5-mM isopropyl-beta-D-thiogalactopyranoside (IPTG) at 16°C for 12 h. Recombinant proteins were purified on a nickel–nitrilotriacetic acid column (Sangon, C600033). The pGEX-4T-PGI expression vectors were transformed into *Escherichia coli* strain BL21 (DE3), and protein expression was induced by 0.5-mM IPTG at 28°C for 4 h. Recombinant proteins were purified on a Mag-Beads GST Fusion Protein Purification (Sangon, C650031).

### Surface plasmon resonance assay

SPR experiments to analyze protein–protein interactions were performed with a Biacore T200 system (GE Healthcare) using an NTA sensor chip (GE Healthcare). For interaction studies, proteins were prepared in SPR running buffer (50-mM HEPES, pH 7.5, 150-mM NaCl, and 0.1% Tween 20). PGI protein was immobilized on the chip; a kinetics protocol using various concentrations of TLP2 ranging from 0 to 400 µM was employed in independent experiments; and the KD values of the PGI-TLP2 interaction were then calculated.

### Detection of PGI activity

PGI activity was determined according to previously described methods (59). For the assay, 190 µL of assay buffer (50-mM HEPES–NaOH, pH 7.4, 1-mM EDTA, 3-mM MgCl_2_, 3-mM Fru6P, 1-mM NAD^+^, and 0.4-unit/mL Fru6P dehydrogenase) was added to 20 µL of protein extracted from the WT strain or from *G. lucidum* transformants. NADH formation was measured by reading the absorbance at 340 nm, and PGI activity was calculated based on the amount of NADH produced, which was normalized to the total protein level.

### Northern blot analysis

Northern blot analysis of total RNA isolated using TRIzol reagent (Takara) was performed. The quality of total RNA was evaluated by the 28S:18S ratio according to gel electrophoresis. The RNA (10 µg per sample) was denatured by glyoxal treatment and separated on a 1% agarose gel. After capillary transfer onto nylon membranes, specific TLP1 and TLP2 probes were labeled in an *in vitro* transcription reaction with digoxigenin-11-UTP using the DIG-High Prime DNA Labeling and Detection Starter Kit II (cat. 11745832910, Roche) according to the manufacturer’s instructions (60). The membrane was hybridized with a denatured DIG-labeled RNA probe overnight at 68°C with gentle agitation. After hybridization, the blots were washed, and immunological detection was performed. The chemiluminescent signal was detected for 30 minutes with a Bio-Rad Image Lab device.

### Statistical analysis

All data presented in this article are based on the results from at least three independent experiments. The error bars indicate the standard deviations of the means of the values obtained in independent experiments conducted in triplicate. Differences in mean values among groups were analyzed by one-way or two-way analysis of variance using GraphPad Prism. For statistical representation, *P < 0.05, **P < 0.01, ***P < 0.001, and ****P < 0.0001 indicate statistical significance. NS indicates “not significant.”

## Data Availability

The raw sequence reads are deposited in the National Center for Biotechnology Information (NCBI) Sequence Read Archive, accession number PRJNA933587. The gene sequence was deposited into the NCBI GenBank under the following accession numbers: PRMT5 (GenBank: OP360010), PMI (GenBank: UOF75523), PGM (GenBank: UOF75521), and PGI (GenBank: UOF75525).

## SUPPLEMENTAL MATERIAL

Supplemental figures spectrum.02906-23-s0001.pdf Fig. S1 to S3.

ASM does not own the copyrights to Supplemental Material that may be linked to, or accessed through, an article. The authors have granted ASM a non-exclusive, world-wide license to publish the Supplemental Material files. Please contact the corresponding author directly for reuse.

## References

[1] Bedford MT , Clarke SG . 2009. Protein arginine methylation in mammals: who, what, and why. Mol Cell 33:1–13. doi:10.1016/j.molcel.2008.12.013 19150423 PMC3372459

[2] Matera AG , Wang ZF . 2014. A day in the life of the spliceosome. Nat Rev Mol Cell Biol 15:108–121. doi:10.1038/nrm3742 24452469 PMC4060434

[3] Koh CM , Bezzi M , Guccione E . 2015. The where and the how of PRMT5. Curr Mol Bio Rep 1:19–28. doi:10.1007/s40610-015-0003-5

[4] Rouhana L , Vieira AP , Roberts-Galbraith RH , Newmark PA . 2012. PRMT5 and the role of symmetrical dimethylarginine in chromatoid bodies of planarian stem cells. Development 139:1083–1094. doi:10.1242/dev.076182 22318224 PMC3283120

[5] Liu H , Ma X , Han HN , Hao YJ , Zhang XS . 2016. AtPRMT5 regulates shoot regeneration through mediating histone H4R3 dimethylation on KRPs and pre-mRNA splicing of RKP in Arabidopsis. Mol Plant 9:1634–1646. doi:10.1016/j.molp.2016.10.010 27780782

[6] Bao SL , Qyang YB , Yang PR , Kim HW , Du HY , Bartholomeusz G , Henkel J , Pimental R , Verde F , Marcus S . 2001. The highly conserved protein methyltransferase, Skb1, is a mediator of hyperosmotic stress response in the fission yeast Schizosaccharomyces pombe. J Biol Chem 276:14549–14552. doi:10.1074/jbc.C100096200 11278267

[7] Lovely CB , Aulakh KB , Perlin MH . 2011. Role of Hsl7 in morphology and pathogenicity and its interaction with other signaling components in the plant pathogen Ustilago maydis. Eukaryot Cell 10:869–883. doi:10.1128/EC.00237-10 21622903 PMC3147425

[8] Xu XD , Chen Y , Li BQ , Tian SP . 2021. Arginine methyltransferase PeRmtC regulates development and pathogenicity of Penicillium expansum via mediating key genes in conidiation and secondary metabolism. J Fungi (Basel) 7:807. doi:10.3390/jof7100807 34682229 PMC8537047

[9] Scaglione A , Patzig J , Liang J , Frawley R , Bok J , Mela A , Yattah C , Zhang J , Teo SX , Zhou T , Chen S , Bernstein E , Canoll P , Guccione E , Casaccia P . 2018. PRMT5-mediated regulation of developmental myelination. Nat Commun 9:2840. doi:10.1038/s41467-018-04863-9 30026560 PMC6053423

[10] Pastore F , Bhagwat N , Pastore A , Radzisheuskaya A , Karzai A , Krishnan A , Li B , Bowman RL , Xiao W , Viny AD , Zouak A , Park YC , Cordner KB , Braunstein S , Maag JL , Grego A , Mehta J , Wang M , Lin H , Durham BH , Koche RP , Rampal RK , Helin K , Scherle P , Vaddi K , Levine RL . 2020. PRMT5 inhibition modulates E2F1 methylation and gene-regulatory networks leading to therapeutic efficacy in JAK2^V617F^-mutant MPN. Cancer Discov 10:1742–1757. doi:10.1158/2159-8290.CD-20-0026 32669286 PMC7642059

[11] Deng XA , Gu LF , Liu CY , Lu TC , Lu FL , Lu ZK , Cui P , Pei YX , Wang BC , Hu SN , Cao XF . 2010. Arginine methylation mediated by the Arabidopsis homolog of PRMT5 is essential for proper pre-mRNA splicing. Proc Natl Acad Sci USA 107:19114–19119. doi:10.1073/pnas.1009669107 20956294 PMC2973915

[12] Li Z , Yu J , Hosohama L , Nee K , Gkountela S , Chaudhari S , Cass AA , Xiao X , Clark AT . 2015. The SM protein methyltransferase PRMT5 is not required for primordial germ cell specification in mice. EMBO J 34:748–758. doi:10.15252/embj.201489319 25519955 PMC4369312

[13] Mulvaney KM , Blomquist C , Acharya N , Li R , Ranaghan MJ , O’Keefe M , Rodriguez DJ , Young MJ , Kesar D , Pal D , Stokes M , Nelson AJ , Jain SS , Yang A , Mullin-Bernstein Z , Columbus J , Bozal FK , Skepner A , Raymond D , LaRussa S , McKinney DC , Freyzon Y , Baidi Y , Porter D , Aguirre AJ , Ianari A , McMillan B , Sellers WR . 2021. Molecular basis for substrate recruitment to the PRMT5 methylosome. Mol Cell 81:3481–3495. doi:10.1016/j.molcel.2021.07.019 34358446 PMC9016627

[14] Huang SA , Balgi A , Pan YP , Li M , Zhang XR , Du LL , Zhou M , Roberge M , Li X . 2016. Identification of methylosome components as negative regulators of plant immunity using chemical genetics. Mol Plant 9:1620–1633. doi:10.1016/j.molp.2016.10.006 27756575

[15] van der Wel H , Loeve K . 1972. Isolation and characterization of thaumatin I and II, the sweet-tasting proteins from Thaumatococcus daniellii Benth. Eur J Biochem 31:221–225. doi:10.1111/j.1432-1033.1972.tb02522.x 4647176

[16] Brandazza A , Angeli S , Tegoni M , Cambillau C , Pelosi P . 2004. Plant stress proteins of the thaumatin-like family discovered in animals. FEBS Lett 572:3–7. doi:10.1016/j.febslet.2004.07.003 15304314

[17] Nawrot R , Musidlak O , Barylski J , Nowicki G , Bałdysz S , Czerwoniec A , Goździcka-Józefiak A . 2021. Characterization and expression of a novel thaumatin-like protein (CcTLP1) from papaveraceous plant Corydalis cava. Int J Biol Macromol 189:678–689. doi:10.1016/j.ijbiomac.2021.08.067 34390750

[18] He L , Li L , Zhu Y , Pan Y , Zhang X , Han X , Li M , Chen C , Li H , Wang C . 2021. BolTLP1, a thaumatin-like protein gene, confers tolerance to salt and drought stresses in broccoli (Brassica oleracea L. var. Italica). IJMS 22:11132. doi:10.3390/ijms222011132 34681789 PMC8537552

[19] Zhu H , Deng M , Yang Z , Mao L , Jiang S , Yue Y , Zhao K . 2021. Two tomato (Solanum lycopersicum) thaumatin-like protein genes confer enhanced resistance to late blight (Phytophthora infestans). Phytopathology 111:1790–1799. doi:10.1094/PHYTO-06-20-0237-R 33616418

[20] Hage H , Miyauchi S , Virágh M , Drula E , Min B , Chaduli D , Navarro D , Favel A , Norest M , Lesage-Meessen L , Bálint B , Merényi Z , de Eugenio L , Morin E , Martínez AT , Baldrian P , Štursová M , Martínez MJ , Novotny C , Magnuson JK , Spatafora JW , Maurice S , Pangilinan J , Andreopoulos W , LaButti K , Hundley H , Na H , Kuo A , Barry K , Lipzen A , Henrissat B , Riley R , Ahrendt S , Nagy LG , Grigoriev IV , Martin F , Rosso M-N . 2021. Gene family expansions and Transcriptome signatures uncover fungal adaptations to wood decay. Environ Microbiol 23:5716–5732. doi:10.1111/1462-2920.15423 33538380 PMC8596683

[21] Sakamoto Y , Watanabe H , Nagai M , Nakade K , Takahashi M , Sato T . 2006. Lentinula edodes tlg1 encodes a thaumatin-like protein that is involved in lentinan degradation and fruiting body senescence. Plant Physiol 141:793–801. doi:10.1104/pp.106.076679 16648221 PMC1475445

[22] Zhang J , Liu Y , Tang Q , Zhou S , Feng J , Chen H . 2019. Polysaccharide of Ganoderma and its bioactivities. Adv Exp Med Biol 1181:107–134. doi:10.1007/978-981-13-9867-4_4 31677141

[23] Wei Z , Liu L , Guo X , Li Y , Hou B , Fan Q , Wang K , Luo Y , Zhong J . 2016. Sucrose fed-batch strategy enhanced biomass, polysaccharide, and ganoderic acids production in fermentation of Ganoderma lucidum 5.26. Bioprocess Biosyst Eng 39:37–44. doi:10.1007/s00449-015-1480-x 26531749

[24] Hu YR , Li MJ , Wang SL , Yue SN , Shi L , Ren A , Zhao MW . 2018. Ganoderma lucidum phosphoglucomutase is required for hyphal growth, polysaccharide production, and cell wall integrity. Appl Microbiol Biotechnol 102:1911–1922. doi:10.1007/s00253-017-8730-6 29349492

[25] Kim JW , Dang CV . 2005. Multifaceted roles of glycolytic enzymes. Trends Biochem Sci 30:142–150. doi:10.1016/j.tibs.2005.01.005 15752986

[26] Limón MC , Pakula T , Saloheimo M , Penttilä M . 2011. The effects of disruption of phosphoglucose isomerase gene on carbon utilisation and cellulase production in Trichoderma reesei Rut-C30. Microb Cell Fact 10:40. doi:10.1186/1475-2859-10-40 21609467 PMC3126698

[27] Upadhyay S , Shaw BD . 2006. A phosphoglucose isomerase mutant in Aspergillus nidulans is defective in hyphal polarity and conidiation. Fungal Genet Biol 43:739–751. doi:10.1016/j.fgb.2006.05.002 16798030

[28] Zhou ZH , Zhang J , Lu F , Duan YB , Zhou MG . 2021. Glucose-6-phosphate isomerase FgGPI, a β2 tubulin-interacting protein, is indispensable for fungal development and deoxynivalenol biosynthesis in Fusarium graminearum. Phytopathology 111:531–540. doi:10.1094/PHYTO-07-20-0279-R 33544003

[29] Ramos A , Boels IC , de Vos WM , Santos H . 2001. Relationship between glycolysis and exopolysaccharide biosynthesis in Lactococcus lactis. Appl Environ Microbiol 67:33–41. doi:10.1128/AEM.67.1.33-41.2001 11133425 PMC92509

[30] Sanfélix-Haywood N , Coll-Marqués JM , Yebra MJ . 2011. Role of α‐phosphoglucomutase and phosphoglucose isomerase activities at the branching point between sugar catabolism and anabolism in Lactobacillus casei. J Appl Microbiol 111:433–442. doi:10.1111/j.1365-2672.2011.05045.x 21605291

[31] Wang Y , Chen J , Han J , Yang Z , Zhu J , Ren A , Shi L , Yu H , Zhao M . 2022. Cloning and characterization of phosphoglucose isomerase in Lentinula edodes. J Basic Microbiol 62:740–749. doi:10.1002/jobm.202100598 35199357

[32] Zhou H , Bi P , Wu X , Huang F , Yang H . 2014. Improved polysaccharide production in submerged culture of Ganoderma lucidum by the addition of coixenolide. Appl Biochem Biotechnol 172:1497–1505. doi:10.1007/s12010-013-0623-2 24222498

[33] Mu D , Shi L , Ren A , Li M , Wu F , Jiang A , Zhao M . 2012. The development and application of a multiple gene co-silencing system using endogenous URA3 as a reporter gene in Ganoderma lucidum. PLoS One 7:e43737. doi:10.1371/journal.pone.0043737 22937087 PMC3427163

[34] Cör D , Knez Ž , Knez Hrnčič M . 2018. Antitumour, antimicrobial, antioxidant and antiacetylcholinesterase effect of Ganoderma lucidum terpenoids and polysaccharides: a review. Molecules 23:649. doi:10.3390/molecules23030649 29534044 PMC6017764

[35] Meister G , Eggert C , Bühler D , Brahms H , Kambach C , Fischer U . 2001. Methylation of SM proteins by a complex containing PRMT5 and the putative U snRNP assembly factor pICln. Curr Biol 11:1990–1994. doi:10.1016/s0960-9822(01)00592-9 11747828

[36] Gonsalvez GB , Tian L , Ospina JK , Boisvert FM , Lamond AI , Matera AG . 2007. Two distinct arginine methyltransferases are required for biogenesis of SM-class ribonucleoproteins. J Cell Biol 178:733–740. doi:10.1083/jcb.200702147 17709427 PMC2064538

[37] Boisvert FM , Côté J , Boulanger MC , Richard S . 2003. A proteomic analysis of arginine-methylated protein complexes. Mol Cell Proteomics 2:1319–1330. doi:10.1074/mcp.M300088-MCP200 14534352

[38] Zhang Z , Zhang S , Zhang Y , Wang X , Li D , Li Q , Yue M , Li Q , Zhang Y , Xu Y , Xue Y , Chong K , Bao S . 2011. Arabidopsis floral initiator SKB1 confers high salt tolerance by regulating transcription and pre-mRNA splicing through altering histone H4R3 and small nuclear ribonucleoprotein LSM4 methylation. Plant Cell 23:396–411. doi:10.1105/tpc.110.081356 21258002 PMC3051234

[39] Kim H , Ronai ZA . 2020. PRMT5 function and targeting in cancer. Cell Stress 4:199–215. doi:10.15698/cst2020.08.228 32743345 PMC7380451

[40] Maron MI , Casill AD , Gupta V , Roth JS , Sidoli S , Query CC , Gamble MJ , Shechter D . 2022. Type I and II PRMTs inversely regulate post-transcriptional intron detention through SM and CHTOP methylation. Elife 11:e72867. doi:10.7554/eLife.72867 34984976 PMC8765754

[41] El-Andaloussi N , Valovka T , Toueille M , Steinacher R , Focke F , Gehrig P , Covic M , Hassa PO , Schär P , Hübscher U , Hottiger MO . 2006. Arginine methylation regulates DNA polymerase beta. Mol Cell 22:51–62. doi:10.1016/j.molcel.2006.02.013 16600869

[42] Stopa N , Krebs JE , Shechter D . 2015. The PRMT5 arginine methyltransferase: many roles in development, cancer and beyond. Cell Mol Life Sci 72:2041–2059. doi:10.1007/s00018-015-1847-9 25662273 PMC4430368

[43] Radzisheuskaya A , Shliaha PV , Grinev V , Lorenzini E , Kovalchuk S , Shlyueva D , Gorshkov V , Hendrickson RC , Jensen ON , Helin K . 2019. PRMT5 methylome profiling uncovers a direct link to splicing regulation in acute myeloid leukemia. Nat Struct Mol Biol 26:999–1012. doi:10.1038/s41594-019-0313-z 31611688 PMC6858565

[44] Sanchez SE , Petrillo E , Beckwith EJ , Zhang X , Rugnone ML , Hernando CE , Cuevas JC , Godoy Herz MA , Depetris-Chauvin A , Simpson CG , Brown JWS , Cerdán PD , Borevitz JO , Mas P , Ceriani MF , Kornblihtt AR , Yanovsky MJ . 2010. A methyl transferase links the circadian clock to the regulation of alternative splicing. Nature 468:112–116. doi:10.1038/nature09470 20962777

[45] Hu J , Yang H , Mu J , Lu T , Peng J , Deng X , Kong Z , Bao S , Cao X , Zuo J . 2017. Nitric oxide regulates protein methylation during stress responses in plants. Mol Cell 67:702–710. doi:10.1016/j.molcel.2017.06.031 28757206

[46] Zhang L , Wan Y , Huang G , Wang D , Yu X , Huang G , Guo J . 2015. The exosome controls alternative splicing by mediating the gene expression and assembly of the spliceosome complex. Sci Rep 5:13403. doi:10.1038/srep13403 26306464 PMC4549623

[47] Thimme Gowda C , Purama SNS , Kammara R . 2020. TLPdb: a resource for thaumatin-like proteins. Protein J 39:301–307. doi:10.1007/s10930-020-09909-w 32696292

[48] Greenstein S , Shadkchan Y , Jadoun J , Sharon C , Markovich S , Osherov N . 2006. Analysis of the Aspergillus nidulans thaumatin-like cetA gene and evidence for transcriptional repression of pyr4 expression in the cetA-disrupted strain. Fungal Genet Biol 43:42–53. doi:10.1016/j.fgb.2005.10.001 16376592

[49] Belaish R , Sharon H , Levdansky E , Greenstein S , Shadkchan Y , Osherov N . 2008. The Aspergillus nidulans cetA and calA genes are involved in conidial germination and cell wall morphogenesis. Fungal Genet Biol 45:232–242. doi:10.1016/j.fgb.2007.07.005 17703972

[50] Singh S , Tripathi RK , Lemaux PG , Buchanan BB , Singh J . 2017. Redox-dependent interaction between thaumatin-like protein and β-glucan influences malting quality of barley. Proc Natl Acad Sci USA 114:7725–7730. doi:10.1073/pnas.1701824114 28634304 PMC5530668

[51] Oh SA , Park HJ , Kim MH , Park SK . 2021. Analysis of sticky generative cell mutants reveals that suppression of callose deposition in the generative cell is necessary for generative cell internalization and differentiation in Arabidopsis. Plant J 106:228–244. doi:10.1111/tpj.15162 33458909

[52] Nishi H , Hashimoto K , Panchenko AR . 2011. Phosphorylation in protein-protein binding: Effect on stability and function. Structure 19:1807–1815. doi:10.1016/j.str.2011.09.021 22153503 PMC3240861

[53] Liu R , Zhu T , Chen X , Wang Z , Yang Z , Ren A , Shi L , Yu H , Zhao M . 2022. GSNOR regulates ganoderic acid content in Ganoderma lucidum under heat stress through S-nitrosylation of catalase. Commun Biol 5:32. doi:10.1038/s42003-021-02988-0 35017648 PMC8752759

[54] Liu R , Zhu T , Yang T , Yang Z , Ren A , Shi L , Zhu J , Yu H , Zhao M . 2021. Nitric oxide regulates ganoderic acid biosynthesis by the S-nitrosylation of aconitase under heat stress in Ganoderma lucidum. Environ Microbiol 23:682–695. doi:10.1111/1462-2920.15109 32483888

[55] DuBois M , Gilles KA , Hamilton JK , Rebers PA , Smith F . 1956. Colorimetric method for determination of sugars and related substances. Anal. Chem 28:350–356. doi:10.1021/ac60111a017

[56] Li MJ , Chen TX , Gao T , Miao ZG , Jiang AL , Shi L , Ren A , Zhao MW . 2015. UDP-glucose pyrophosphorylase influences polysaccharide synthesis, cell wall components, and hyphal branching in Ganoderma lucidum via regulation of the balance between glucose-1-phosphate and UDP-glucose. Fungal Genet Biol 82:251–263. doi:10.1016/j.fgb.2015.07.012 26235043

[57] Tian JL , Ren A , Wang T , Zhu J , Hu YR , Shi L , Yu HS , Zhao MW . 2019. Hydrogen sulfide, a novel small molecule signalling agent, participates in the regulation of ganoderic acids biosynthesis induced by heat stress in Ganoderma lucidum. Fungal Genet Biol 130:19–30. doi:10.1016/j.fgb.2019.04.014 31028914

[58] Chan-Penebre E , Kuplast KG , Majer CR , Boriack-Sjodin PA , Wigle TJ , Johnston LD , Rioux N , Munchhof MJ , Jin L , Jacques SL , West KA , Lingaraj T , Stickland K , Ribich SA , Raimondi A , Scott MP , Waters NJ , Pollock RM , Smith JJ , Barbash O , Pappalardi M , Ho TF , Nurse K , Oza KP , Gallagher KT , Kruger R , Moyer MP , Copeland RA , Chesworth R , Duncan KW . 2015. A selective inhibitor of PRMT5 with in vivo and in vitro potency in MCL models. Nat Chem Biol 11:432–437. doi:10.1038/nchembio.1810 25915199

[59] Mahanty S , Madrid EM , Nash TE . 2013. Quantitative screening for anticestode drugs based on changes in baseline enzyme secretion by Taenia crassiceps. Antimicrob Agents Chemother 57:990–995. doi:10.1128/AAC.01022-12 23229489 PMC3553704

[60] Zhu Q , Ding L , Zi Z , Gao S , Wang C , Wang Y , Zhu C , Yuan Z , Wei F , Cai Q . 2019. Viral-mediated AURKB cleavage promotes cell segregation and tumorigenesis. Cell Rep 27:1633–1636. doi:10.1016/j.celrep.2019.04.057 31042486

