# Supplementary material for: PRMT5 regulates the polysaccharide content by controlling the splicing of thaumatin-like protein in Ganoderma lucidum
Source: Microbiol Spectr. 2023 Oct 26;11(6):e02906-23. doi: 10.1128/spectrum.02906-23 (PMC10715077; doi:10.1128/spectrum.02906-23)
Supplement: Supplemental figures — Fig. S1 to S3. [file spectrum.02906-23-s0001.pdf]

SUPPLEMENTAL MATERIAL

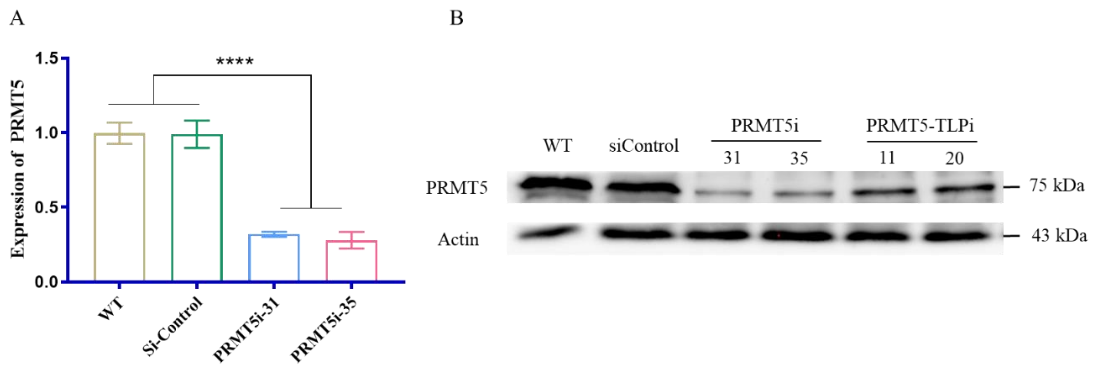

Fig. S1 Assessment of the efficiency of *PRMT5* silencing

A. The *PRMT5* gene expression levels of the candidate strains were measured by qRT-PCR. B. The expression of the PRMT5 protein was confirmed by Western blot analysis with an anti-PRMT5 antibody. β-Actin was used as an internal reference for data normalization. The data show the means ± SDs of the values obtained from three independent experiments (*****P* < 0.0001 by one-way ANOVA).

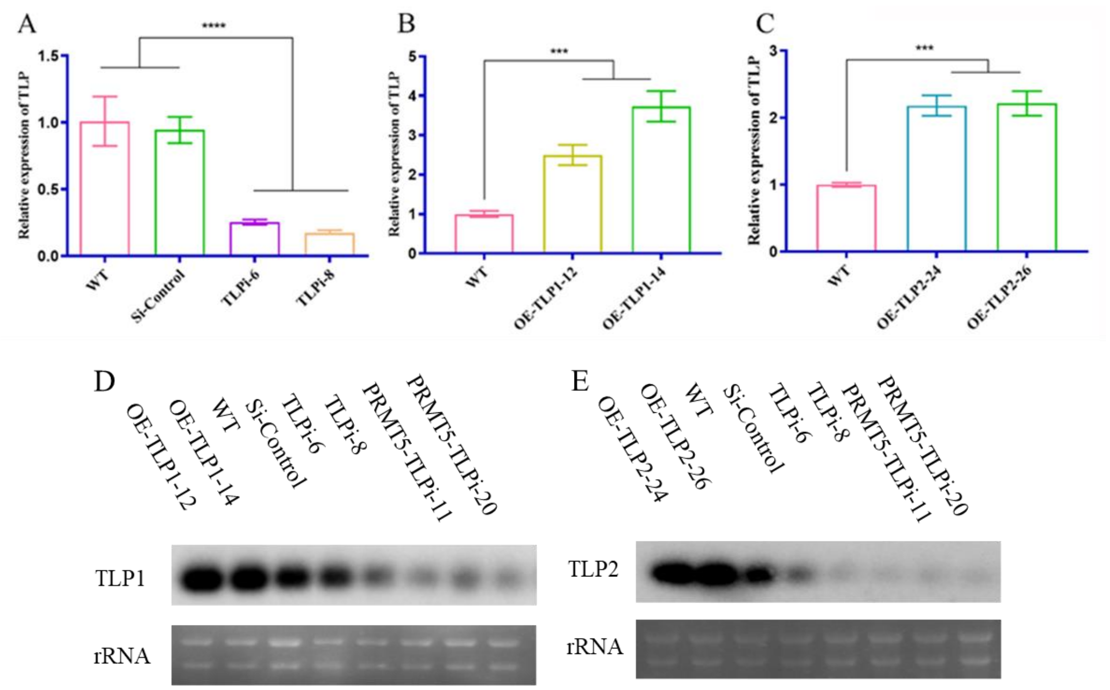

Fig. S2 Assessment of the efficiency of *TLP* silencing and overexpression

A. The *TLP* expression levels of the silenced candidate strains were measured by qRT-PCR, two *TLP*-silenced strains (*TLP*i-6 and *TLP*i-8). B. The *TLP* expression levels of the *TLP1*-overexpressing candidate strains were measured by qRT-PCR, two longer *TLP* isoform-overexpressing strains (OE-*TLP1*-12 and OE-*TLP1*-14). C. The *TLP* expression levels of the *TLP2*-overexpressing candidate strains were measured by qRT-PCR, two shorter *TLP* isoform-overexpressing strains (OE-*TLP2*-24 and OE-*TLP2*-26). D. Northern blot analysis of *TLP1* expression in the WT, *TLP*-silenced and *TLP1*-overexpressing strains. The panels show the Northern blot hybridized with the specific TLP1 probes indicated, and the gel loading controls are shown with the rRNAs in the bottom panel. E. Northern blot analysis of *TLP2* expression in the WT, *TLP*-silenced and *TLP2*-overexpressing strains. The panels show the Northern blot hybridized with specific TLP2 probes indicated, and the gel loading controls are shown with the rRNAs in the bottom panel. The data show the means ± SDs of the values obtained from three independent experiments (***$P < 0.001$, ****$P < 0.0001$ by one-way ANOVA).

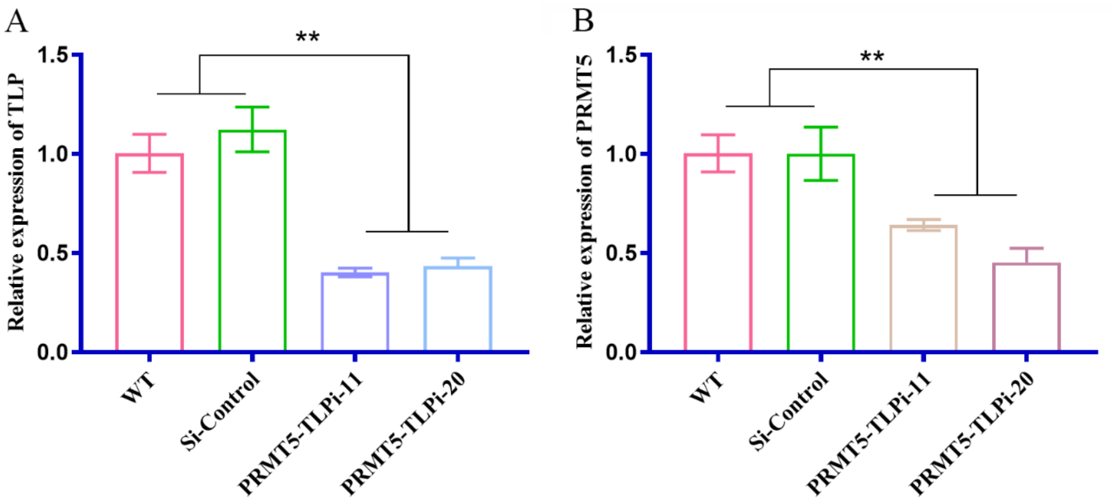

Fig. S3 Assessment of the efficiency of *PRMT5* and *TLP* silencing in the *PRMT5-TLP*-cosilenced strains

A. PRMT5 expression in the *PRMT5*-and-*TLP*-cosilenced candidate strains was measured by qRT-PCR. B. The expression levels of *TLP* in the *PRMT5*-and-*TLP* -cosilenced candidate strains were measured by qRT-PCR. The data are presented as the means ± SDs of the values obtained from three independent experiments (***P* < 0.01 by one-way ANOVA).
